# A systematic review of risk stratification tools internationally used in primary care settings

**DOI:** 10.1002/hsr2.329

**Published:** 2021-07-23

**Authors:** Shelley‐Ann M. Girwar, Robert Jabroer, Marta Fiocco, Stephen P. Sutch, Mattijs E. Numans, Marc A. Bruijnzeels

**Affiliations:** ^1^ Department of Public Health and Primary Care, LUMC Campus the Hague Leiden University Medical Centre The Hague The Netherlands; ^2^ Jan van Es Instituut Ede The Netherlands; ^3^ Mathematical Institute Leiden University Leiden The Netherlands; ^4^ Medical Statistics Department of Biomedical Data Science Leiden University Medical Center Leiden The Netherlands; ^5^ Princess Maxima Center for Pediatric Oncology Utrecht The Netherlands; ^6^ Department of Health Policy and Management Bloomberg School of Public Health Johns Hopkins University Baltimore Maryland USA

**Keywords:** population health management, primary healthcare, risk assessment

## Abstract

**Background and Aims:**

In our current healthcare situation, burden on healthcare services is increasing, with higher costs and increased utilization. Structured population health management has been developed as an approach to balance quality with increasing costs. This approach identifies sub‐populations with comparable health risks, to tailor interventions for those that will benefit the most. Worldwide, the use of routine healthcare data extracted from electronic health registries for risk stratification approaches is increasing. Different risk stratification tools are used on different levels of the healthcare continuum. In this systematic literature review, we aimed to explore which tools are used in primary healthcare settings and assess their performance.

**Methods:**

We performed a systematic literature review of studies applying risk stratification tools with health outcomes in primary care populations. Studies in Organisation for Economic Co‐operation and Development countries published in English‐language journals were included. Search engines were utilized with keywords, for example, “primary care,” “risk stratification,” and “model.” Risk stratification tools were compared based on different measures: area under the curve (AUC) and C‐statistics for dichotomous outcomes and *R* ^2^ for continuous outcomes.

**Results:**

The search provided 4718 articles. Specific election criteria such as primary care populations, generic health utilization outcomes, and routinely collected data sources identified 61 articles, reporting on 31 different models. The three most frequently applied models were the Adjusted Clinical Groups (ACG, n = 23), the Charlson Comorbidity Index (CCI, n = 19), and the Hierarchical Condition Categories (HCC, n = 7). Most AUC and C‐statistic values were above 0.7, with ACG showing slightly improved scores compared with the CCI and HCC (typically between 0.6 and 0.7).

**Conclusion:**

Based on statistical performance, the validity of the ACG was the highest, followed by the CCI and the HCC. The ACG also appeared to be the most flexible, with the use of different international coding systems and measuring a wider variety of health outcomes.

## 1 INTRODUCTION

For several decades, healthcare costs have been rising. This has been attributed to aging populations and innovative ways of curing and treating diseases, leading to an increased prevalence of chronic illnesses and comorbidities among community dwelling older people.^1^ Also patients have increased demands regarding increasing choice around the way their healthcare should be organized and have tended to utilize more care. Furthermore, the needs for healthcare are not evenly distributed within populations. In Western countries, the sickest 5% of the population make up for 50% of the total healthcare costs.^2^ In order to maintain high‐quality healthcare, resources should be distributed according to the needs of the population instead of the demand. One way of dealing with this is to allocate resources according to the individual care needs in subpopulations. Predicting healthcare utilization and health outcomes based on needs provides opportunities to allocate resources more appropriately. Predictions of health outcomes through risk stratification can be used to tailor proactive clinical care, to install preventive measures, to restructure healthcare, and to improve insight for healthcare professionals. In the long run, this approach will help improve the quality of care and reduce the costs.^3^, ^4^

A way to monitor and predict costly patient outcomes, such as hospitalization, high‐care utilization, and emergency department visits, is through the use of structured population health management programs. Population health management is an approach that aims to improve the health of a defined group of people and to strive for more equitable distribution of health outcomes within the group. In population health management programs, an important step is to stratify individuals within a specific subpopulation according to the risk of experiencing an adverse event, such as defined undesirable health outcomes or the extent of their healthcare utilization. Stratification analyses are often performed based on the use of routinely collected healthcare data. Typically, the high‐risk sub‐population usually comprises of a small percentage of the total population. The medium‐ and low‐risk subpopulations are much larger with around 35% of the overall population classified as medium risk and 60% as low risk.^2^ The identification of people classified on their respective risk estimates is referred to as risk stratification. Preceding risk stratification population segmentation is performed. Segmentation can be performed based on general characteristics such as age, gender, and specific diseases but also on morbidity and healthcare utilization patterns. A discussion of segmentation was outside the scope of this study.

Many methods for risk stratification exist internationally. Current literature regarding risk stratification models prominently focuses on stratifying hospital populations, based on readily available hospital data. However, primary care data have a great potential to improve healthcare quality and reduce health costs.^5^ Especially, in countries where primary care registries have nearly 100% coverage of the total population, such as the Netherlands and the United Kingdom (UK), the opportunity arises to assess the whole population by using these routinely collected primary care data. Distribution of risk in a primary care population is different from a hospital or specialized care population. Current literature also mainly focuses on risk stratification models with disease‐specific outcomes, whereas in this study. The focus is on more generic utilization outcomes such as risk on hospitalization, emergency department visits, future high healthcare utilization, and high pharmaceutical expenditures.

The aim of this study was to perform a systematic literature review to describe and assess the performance of different risk stratification tools with generic health utilization outcomes using routinely collected data and with possibilities of application to the European context, such as in Dutch primary care. Based on the description of the performance of the tools, we recommend the risk stratification tool best suited for usage in Dutch primary care.

## 2 METHODS

The PRISMA statements regarding conduction and reporting systematic literature reviews were followed throughout the literature review process.^6^

This review was conducted through searches in the search engines Pubmed and Embase. The search‐string which contained both keywords and MeSH terms is shown in the Supporting Information S1. The most important keywords were “primary care,” “risk stratification,” and “model.” EndNote X8.2 was used as the reference manager for the articles. The search‐string was produced in collaboration with the Leiden University Medical Center (LUMC) Walaeus library.

**FIGURE 1:**
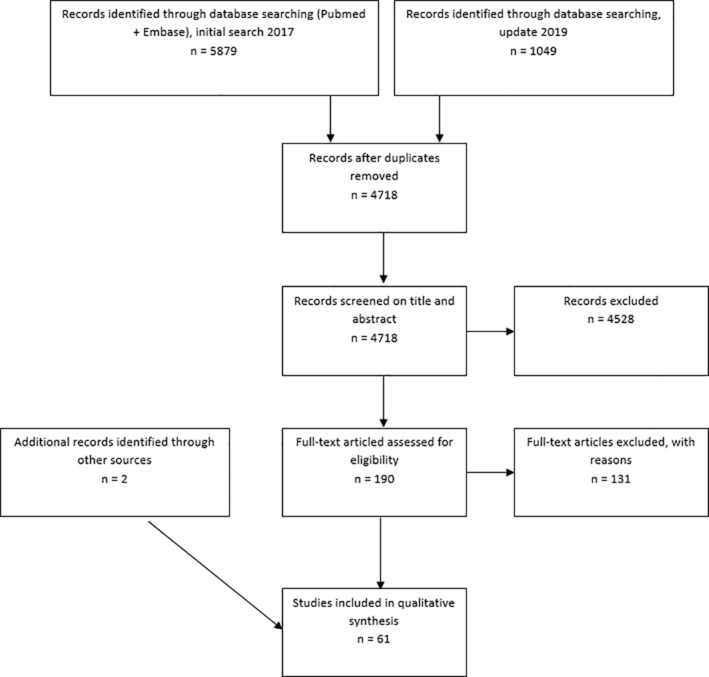
PRISMA flowchart displaying numbers of included and excluded articles

The PRISMA flow diagram displays the numbers of included and excluded articles (Figure 1).

### 2.1 Inclusion criteria

The search characteristics are specified by the *P*opulation, *I*ntervention, *C*ontrol, and *O*utcome method. In our research, the *population* is the primary care population. Therefore, we only included articles where models applied to primary care populations are discussed. The *interventions* investigated were the risk stratification approaches and models that are applied to primary care data. *Outcomes* investigated are risks of hospitalization, high healthcare costs, emergency department visits, high pharmaceutical drug expenditure, mortality, and other generic health utilization outcomes.

For comparability with a Western‐societal environment such as the Dutch situation, only studies performed in countries listed with the Organisation for Economic Co‐operation and Development,^7^ were included. Only freely accessible articles in the English language were considered eligible. Articles from January 2007 till August 2019 were reviewed. The inclusion criteria narrowed the search down to a context which was more applicable in a European primary care situation with a gatekeeper's role, such as the Dutch primary care system.

### 2.2 Exclusion criteria

Articles that used risk stratification tools on populations consisting of hospitalized patients or patients seeking consultation with a specialist (eg, an oncologist or cardiologist) were excluded. These patients were not considered to represent those in a primary care setting. In addition, research looking at specific disease outcome was also excluded, as this review aims at exploring general population outcomes. Articles not freely accessible were excluded as well as articles that were not available in English.

The initial search, conducted in December 2017, yielded 5879 articles. In September 2019, an update of the search was conducted, resulting in an additional 1049 articles. After removing duplicates according to the manual of the Free University (VU) library,^8^ 4718 articles remained. Articles were screened on both the title and abstract, based on the criteria mentioned earlier. Seventy‐eight percentage of the screening, based on the title and abstract, was performed by two researchers independently (R.J. and S.G.). Their results were compared, and in the case of disagreement (2%), the articles were discussed until consensus was achieved. The main causes for disagreement concerned indistinct and misunderstood study populations and model outcomes. As the percentage of disagreement was low, the remaining 22% of the titles and abstracts were only screened by one researcher. After screening the title and abstract, 190 articles remained to be screened on their full text. Screening of all 190 full papers was performed by the same two researchers independently, and results were compared. Again, in case of disagreement (21%), the article was discussed until consensus was achieved. After exclusion of 131 articles, including 17 titles which were either not freely accessible or where no English versions of the full papers were available, 59 studies remained to be included in this review. Two further articles were added through the snowball method, resulting in 61 articles.

### 2.3 Assessing performance of models

The different models were compared on three aspects: frequency of use, statistical diagnostic validity, and performance in primary care.

For each identified risk stratification model, *the frequency of use* of the model was presented, taking into account all included studies.

For the assessment of the *statistical diagnostic validity*, reviewed studies were divided into *application*, *validation*, and *comparison* studies. In the *application* studies, risk stratification tools were applied for purposes other than assessing their statistic diagnostic validity. Therefore, *application* studies did not present any statistical diagnostic measures of the risk stratification tools. In the *validation* studies and in most of the *comparison* studies, statistical diagnostic measures of the applied risk stratification tools were provided. Area under the curve (AUC) and C‐statistics for models with dichotomous outcomes and *R* ^2^ values for models with continues outcomes were used to validate risk stratification tools. Models with AUC or C‐statistic values between 0.5 and 0.6 were classified as performing *poorly*, values between 0.6 and 0.7 were considered *sufficient*, and values above 0.7 were considered *good*.^9^ Ten of the reviewed papers, the *comparison* studies, compared more than one risk stratification tool in the same study population with the same record data, enabling a more appropriate comparison between risk stratification tools. Most of the comparison studies presented statistical diagnostic values, as they are mostly also validation studies.

For *performance in primary care*, we assessed the type of routinely collected data that are used as input of the model. Models using input data available in Dutch primary care health records were assumed to have a good potential performance in Dutch primary care.

## 3 RESULTS

A total of 31 risk stratification models were identified in the literature. The three most frequently applied tools, taking into account all included studies, concern the Adjusted Clinical Groups (ACG), the Charlson Comorbidity Index (CCI), and the Hierarchical Condition Categories (HCC). These three main risk stratification tools are presented in Table 1, with predicted outcomes and diagnostic values. Assessment of these tools, their diagnostic validity, and applicability in primary care are described in order. The remaining 28 risk stratification tools can be found in the Supporting Information S2.

**TABLE 1:** Overview of the three most frequently identified risk stratification models with their characteristics and diagnostic properties for different outcomes

	First author, year	Adjusted Clinical Group (ACG)	Charlson Comorbidity Index (CCI)	Hierarchical Condition Categories (HCC)
Categories		ACG categories (1‐93), Resource Utilization Bands (RUBs), Expended Diagnosis Clusters (EDC) count	Six categories based on chronic condition count	Score based on aggregated conditions (70 categories)
Total number of studies in which the model was applied		n = 23	n = 19	n = 7
Diagnostic properties for different outcomes:				
*Hospitalization*	Haas^4^	*C* = 0.73	*C* = 0.68	*C* = 0.67
	Lemke^12^	AUC = 0.80	AUC = 0.78	
*(Number of hospitalizations)*	Shadmi^16^	*R* ^2^ = .24	*R* ^2^ = .11	
*(Unplanned hospitalizations)*	Maltenfort^11^	AUC = 0.82		
	Inouye^20^		*C* = 0.72	
	Ou^21^		*C* = 0.61	
	Mosley^25^			AUC = 0.64
*Emergency department visits*	Haas^4^	*C* = 0.67	*C* = 0.59	*C* = 0.58
	Ou^21^		*C* = 0.63	
	Wallace^22^		*C* = 0.58	
*Costs*				
*(Top 10% total costs)*	Haas^4^	*C* = 0.76	*C* = 0.70	*C* = 0.70
*(Total costs)*	Brilleman^14^	*R* ^2^ = .41	*R* ^2^ = .34	
*(Pharmaceutical costs)*	Aguado^10^	*R* ^2^ = .39		
*(Total costs)*	Sicras‐Mainar^13^	*R* ^2^ = .37		
*(Total costs)*	Charlson^18^		*R* ^2^ = .22	
*(Total costs)*	Charlson^19^		*R* ^2^ = .20	
*(High total costs)*	Ou^21^		*C* = 0.64	
*Utilization of different healthcare services*				
*(GP visits)*	Brilleman^15^	*R* ^2^ = .37	*R* ^2^ = .26	
*(Primary care visits)*	Shadmi^16^	*R* ^2^ = .54	*R* ^2^ = .18	
*(Specialist visits)*	Shadmi^16^	*R* ^2^ = .45	*R* ^2^ = .13	
*(Number of diagnostic imaging tests)*	Shadmi^16^	*R* ^2^ = .37	*R* ^2^ = .15	
*(Visits)*	Sicras‐Mainar^13^	*R* ^2^ = .42		
*(Number of diagnoses/reasons for visit)*	Sicras‐Mainar^13^	*R* ^2^ = .77		
*(High outpatient visits)*	Ou^21^		*C* = 0.63	
Input data for the model		Age, gender, diagnostic codes, pharmaceutical information, healthcare costs	Presence or absence of chronic conditions based on diagnosis codes; weighted	ICD‐9 of ICD‐10 diagnosis codes

Abbreviations: AUC, area under the ROC curve; C, C‐statistic; R^2^, R‐square.

### 3.1 Adjusted clinical groups: 23 Studies

The ACG is the most frequently applied risk stratification tool in our review. The ACG system is a risk stratification model designed by the Johns Hopkins University. The model was originally developed to predict and measure multimorbidity in a population. The ACG system is a measure of comorbidity and can predict utilization costs, hospitalization, and emergency department visits. The model is able to use patients' data from electronic health records (EHRs), insurance claims, disease registries, and health status surveys.^10^ Minimal input data for the model are healthcare diagnoses in a specific time interval, gender, and age, to which the ACG classifies people to one of 93 ACG categories. These categories represent expected healthcare utilization. In addition, different probabilities for future utilization of healthcare services are calculated. This information can be used by healthcare professionals to make informed clinical and administrative decisions.^4^

Of the 23 ACG studies, eight provided statistical diagnostic values for the accuracy of the model, calculated for different outcomes. For *prediction of hospitalization*, the model is diagnostically assessed three times with AUC and C statistic values between 0.73 and 0.82.^4^, ^11^, ^12^ The diagnostic accuracy can be classified as *good*.

In one study, a C‐value of 0.67 is presented for *prediction of emergency department visitation*, which classifies as *sufficient*, and a C‐value of 0.76 for *prediction of high total costs*, again classifying as *good*.^4^ Three other studies presented *R* ^2^ values between 0.37 and 0.41 for explaining the variation of healthcare *costs* by the ACG model.^10^, ^13^, ^14^ *Variations in high utilization of different healthcare services*, such as primary care visits, specialists' visits and numbers of diagnostic imaging tests, diagnoses, and hospitalizations, are discussed in three studies, with *R* ^2^ values ranging from 0.24 to 0.77.^13^, ^15^, ^16^

ACG is highly suitable for application in primary care populations, as using *International Classification of Primary Care* (ICPC) codes as input is possible.^10^ ICPC codes are used to classify complaints and diagnoses of patients in many primary care settings, such as in the Netherlands. This information is stored in EHRs. The model uses other input variables such as age, gender, pharmaceutical information, and previous visitation, stored in the EHR as well.

### 3.2 Charlson comorbidity index: 19 Studies

The CCI is the second‐most studied risk stratification model. The CCI was developed by Charlson and colleagues in 1987 and was originally an age‐comorbidity index that predicted a relative risk of death within a year for hospital‐admitted cancer patients.^17^ Since that time, many adjustments have been made, and in addition to mortality predictions, the model is now used to predict hospitalization, emergency department visitation, future healthcare utilization, and morbidity in wider populations. The system categorizes the population into six categories, based on the presence of comorbidities and chronic conditions, of which a weighted sum is provided (from zero conditions as category 1‐5 or more conditions as category 6).^18^, ^19^ The model investigates the effect of multimorbidity and predicts several outcomes. Variations of the CCI exist, and the validity on predictions has been consistently investigated.^4^

From the 18 studies in which the CCI or a modification was used, 10 provided statistical diagnostic values. AUC and C‐values range from 0.61 to 0.78 for the prediction of future *hospitalization*,^4^, ^12^, ^20^, ^21^ which corresponds to an accuracy of *sufficient* and *good*. For *emergency department visitation*, C‐statistics between 0.58 and 0.63 is provided ^4^, ^21^, ^22^ (*poor* to *sufficient*) and for total *costs*, *R* ^2^ values were between 0.20 and 0.34.^14^, ^18^, ^19^ For *healthcare utilization of different healthcare services*, *R* ^2^ values were between 0.13 and 0.26.^15^, ^16^, ^23^

Input variables for the CCI include combinations of age, race, gender, mental illness, pregnancy, drug or alcohol addiction, type of health plan, type of provider, number of therapeutic classes, and number of medications prescribed. The CCI is fit for use with primary care data but focuses primarily on the absence or presence of chronic conditions, apart from other demographics. Although there is no evidence in the included studies of use of the CCI with ICPC codes, the coding system used in Dutch primary care, there is evidence for use with Read codes, a British primary care coding system.^24^ Possibilities to use the model with coding systems other than International Classifications of Disease (ICD) codes are therefore very likely.

The software algorithm for CCI is published and available.^4^

### 3.3 Hierarchical condition categories: Seven studies

The third most frequently studied model (n = 7) is the HCC. This model was first designed and implemented by the Centers for Medicare and Medicaid Services (CMS) to adjust capitation payments for enrolees with higher risk than others. The model uses demographic data of patients as well as ICD 10th revision (ICD‐10) diagnosis codes. ICD codes are used in all American healthcare service providers.^25^ The ICD classification is adapted in other countries, yet these are codes most prominently used in hospital administrative registries.^26^ Based on this information, the model categorizes a patient into one of 70 aggregated condition categories, which contributes to an individualized risk score.

For this model, four diagnostic values are provided in two studies included in this literature review. For *hospitalization*, an AUC value of 0.64,^25^ and a C‐statistic of 0.67^4^ are provided. The study by Haas et al. provides a C‐statistic equal to 0.58 for prediction of *emergency department visitation*, but a much higher C‐statistic of 0.70 for prediction of *high total costs*.^4^

A major concern regarding this model is that it makes use of ICD codes rather than ICPC codes, making it difficult to apply in the Dutch primary care settings.

### 3.4 Comparison studies

A total of 10 papers compared more than one risk stratification tool applied within the same study populations. However, only five articles compared more than one of the three above‐mentioned risk stratification tools while providing statistical diagnostic values to compare the different tools with each other.

For *hospitalization*, the ACG performs slightly better than the CCI with AUC values of 0.80 vs 0.78^12^ and C‐statistics of 0.73 vs 0.68.^4^ The ACG also outperforms the CCI regarding *emergency department visitation* with C‐statistics of 0.67 vs 0.59 and *high total costs* with C‐statistics of 0.76 vs 0.70,^4^ and *R* ^2^ values of .41 vs .34.^14^ Furthermore, the study by Shadmi and colleagues showed evidence of the ACG providing better results compared to the CCI regarding other *healthcare utilization* outcomes, such as numbers of hospitalizations (*R* ^2^ = .24 vs *R* ^2^ = .11), primary care visits (*R* ^2^ = .54 vs *R* ^2^ = .18), specialist visits (*R* ^2^ = .45 vs *R* ^2^ = .13), and diagnostic imaging tests (*R* ^2^ = .37 vs *R* ^2^ = .15) all within a study period of 12 months.^16^ In addition, Brilleman and colleagues find *R* ^2^ values of .37 for the ACG and 0.26 for the CCI with the number of general practitioner visits as the predicted outcome.^15^

### 3.5 Remaining risk stratification tools

In addition to the three above‐mentioned risk stratification tools, 28 other tools were identified within this systematic literature review. One of the 28 identified risk stratification tools is called the Elixhauser Index or Method and was mentioned in five studies. The Elixhauser Index uses a set of 30 dichotomous variables as comorbidity measures.^27^ Outcomes concern high utilization and pharmaceutical expenditure. One out of the five studies, mentioning the Elixhauser Index, provided C‐statistics between 0.62 and 0.74 for different health utilization outcomes.^21^ The study by Ou and colleagues compared those C‐statistics to values between 0.61 and 0.64 for the CCI.^21^

A number of the identified risk stratification tools include disease or medication counts as comorbidity measures, such as the Chronic Disease Score (CDS; n = 3), which is based on dispensed drugs history. The previously mentioned study by Ou and colleagues provided C‐statistic values between 0.61 and 0.72 for the CDS.^21^ The remainder of the identified risk stratification tools were only mentioned a few times (n = 1, 2, or 3) in the articles, typically including only one validation study per risk stratification tool. The infrequent use of these tools does not make a review possible. The Clinical Risk Groups (CRG), for example, emerged three times within our systematic review. However, all studies using the CRG as a risk stratification tool were application studies and thus lacking statistical diagnostic values. Most other studies describe a new risk stratification tool developed for a specific situation. In Supporting Information S2, all of the risk stratification tools are presented and organized by included studies.

## 4 DISCUSSION

### 4.1 Summary of main findings

This literature review revealed a broad range of risk stratification tools that have been assessed on accuracy and validity. The most common predicted outcomes were future hospitalization, emergency department visitation, high healthcare utilization, and total cost. The three most frequently studied risk stratification tools were the ACG, CCI, and HCC.

With most AUC and C‐statistic values above 0.70, the ACG performs *good* on a wide variety of outcomes. The CCI scores *sufficient* for different outcomes, with the exception of high utilization of healthcare for which a low score yielded. With most AUC and C‐statistic values between 0.60 and 0.70, the HCC can also be classified as *sufficient*. Comparing the results of the ACG, the CCI, and HCC, more convincing evidence for accuracy and validity is found for the ACG. Previous research also indicated the high accuracy and validation of the ACG model.^12^, ^28^, ^29^, ^30^ The model is considered one of the leading models regarding the accuracy of predicting hospitalizations^12^ and is widely used to gain insight in future healthcare utilization of patients.^31^ The study by Ou and colleagues is making a compelling case for the validity of the Elixhauser Index and the CDS, compared to the CCI.^21^ However, this result is not robust as it is only based on a single study. Nevertheless, the Elixhauser Index may have future potential for use in a European primary care setting.

For the applicability in primary care, evidence shows that the ACG has the possibility to make use of ICPC codes, the coding system of the (Dutch) primary care registry. The CCI has not yet been proven usable with ICPC codes. Nevertheless, evidence has shown possibilities for the CCI to be used with Read codes,^24^ the UK's primary care coding system, making it highly likely that the CCI can be applied using other than ICD diagnosis codes. For the HCC model on the other hand, there is no evidence to use diagnosis codes other than the ICD coding system, making it difficult to use this model in Dutch primary care.

The results of this study support the idea that risk stratification tools are suitable for primary care data in a European context. However, different models emphasize various aspects within the tools. As all applications are focusing on similar utilization outcomes, such as hospitalization, ED visits, and costs, the ACG has an array of other indicators developed for risk stratification. Various applications in primary care show the potential of models, for example, in areas of improved resource allocation,^32^ and care management due to better insights into vulnerable populations.^33^ In addition, the ACG provides possibilities to efficiently prioritize sub‐populations for tailored care interventions.^34^

### 4.2 Limitations

Although our results support risk stratification using the ACG in primary care, there are some limitations.

We only selected studies that already performed risk stratification in primary care. As a consequence, we could have missed stratification tools only applied in hospital or open source data but with a strong potential for suitability in primary care.

Selection of studies was dependant on the interobserver reliability of the two researchers. Although inclusion and exclusion criteria were clearly formulated beforehand, the possibility remains that useful tools were missed given the relatively high number of disagreements.

We assessed the identified risk stratification tools in different studies, in an attempt to compare the statistical validity of the models with each other. However, the incomparable circumstances under which different studies are performed, such as study populations and data sources, make reasonable comparisons challenging.

We based our recommendation on diagnostic values of applied risk stratification tool reported by studies published in scientific literature. Due to publication bias, promising risk stratification tools may not have emerged sufficiently from our findings.

### 4.3 Further research

From all the articles included in this study, a small percentage explicitly defines “risk stratification.” With the growing need for tailored care and health management approaches, a precise definition will be useful. Risk stratification and other terms such as population segmentation are now used interchangeably. Studies contributing to a generalized definition of the term *risk stratification* will be of great scientific and practical value. By using the same definition, miscommunications regarding the meaning of risk stratification will be reduced, and information on highly performing methods and implementations thereof can be shared more effectively.

With this review, we studied which risk stratification tools are best suited for the European primary care setting. However, primary care settings differ between countries. To find the best suitable tool for a specific primary care system, the performance of different tools should be investigated within the same setting, centered on desired outcomes. Based on the results of this literature review, further studies assessing the performance of desired risk stratification models will be beneficial for Dutch primary care.

## 5 CONCLUSION

In conclusion, based on application frequency, statistical validity, and used diagnosis coding systems, we suggest the ACG as the best model for use in European primary care settings, such as Dutch Primary Care. However, further local assessment of the ACG system is needed to ensure proper implementation.

## CONFLICT OF INTEREST

All authors declare that there is no conflict of interest that could be perceived as prejudicing the impartiality of the research reported.

## AUTHOR CONTRIBUTIONS

Conceptualizing: Shelley‐Ann Girwar, Robert Jabroer, Marc Bruijnzeels

Data Curation: Shelley‐Ann Girwar, Robert Jabroer

Funding Acquisition: Marc Bruijnzeels

Investigation: Shelley‐Ann Girwar, Robert Jabroer.

Methodology: Shelley‐Ann Girwar, Robert Jabroer, Marc Bruijnzeels

Supervision: Marc Bruijnzeels, Mattijs Numans

Visualization: Shelley‐Ann Girwar, Robert Jabroer, Stephen Sutch, Marta Fiocco, Marc Bruijnzeels

Writing—Original Draft Preparation: Robert Jabroer, Shelley‐Ann Girwar

Writing—Review & Editing: Shelley‐Ann Girwar, Robert Jabroer, Stephen Sutch, Marta Fiocco, Marc Bruijnzeels, Mattijs Numans.

All authors have read and approved the final version of the manuscript.

## TRANSPARENCY STATEMENT

Authors affirm that the manuscript is an honest, accurate and transparent account of the study being reported, that no important aspects of this study have been omitted and that there were no discrepancies from the study as planned.

## ETHICS STATEMENT

Ethical approval was not required for this study.

## Supporting information


**Appendix S1.** Supporting Information.

**Appendix S2.** Supporting Information.

## Data Availability

Data sharing not applicable to this article as no new data were created or analyzed during this study.
