# Supplementary material for: A systematic review of risk stratification tools internationally used in primary care settings
Source: Health Sci Rep. 2021 Jul 23;4(3):e329. doi: 10.1002/hsr2.329 (PMC8299990; doi:10.1002/hsr2.329)
Supplement: Supplementary file 1 — Appendix S1. Supporting Information. [file HSR2-4-e329-s002.docx]

# Supporting Information file 1: Search strategy

(((("risk"[tw] OR "risks"[tw] OR "Risk"[Mesh]) AND ("stratification"[tw] OR "stratifications"[tw] OR "stratified"[tw] OR "stratify"[tw] OR "stratifies"[tw] OR "stratifying"[tw])) AND ("model"[tw] OR "models"[tw] OR "tool"[tw] OR "tools"[tw] OR "method"[tw] OR "methods"[tw] OR "measure"[tw] OR "measures"[tw] OR "measured"[tw] OR "measuring"[tw] OR "algorithm"[tw] OR "algorithms"[tw] OR "metric"[tw] OR "metrics"[tw] OR "score"[tw] OR "scores"[tw] OR "scoring"[tw] OR "index"[tw] OR "indexes"[tw] OR "indices"[tw] OR "indexed"[tw] OR "count"[tw] OR "counts"[tw])) OR "adjusted clinical groups"[tw] OR "Minnesota tiering"[tw] OR "Hierarchical Condition Categories"[tw] OR "elder risk assessment index"[tw] OR "chronic condition count"[tw] OR "Charlson comorbidity index"[tw] OR "chronic disease score"[tw]) AND ("General Practitioners"[Mesh] OR "General Practitioner"[tw] OR "General Practitioners"[tw] OR "physician"[tw] OR "physicians"[tw] OR "general practice"[tw] OR "General Practice"[Mesh] OR "GP"[ti] OR "GPs"[ti] OR "GP's"[ti] OR "Primary Health Care"[Mesh] OR "Primary Health Care"[tw] OR "primary care"[tw] *OR "Family doctor"[tw] OR "family doctors"[tw] OR "Family practice"[tw] OR "Family practices"[tw] OR "Family medicine"[tw] OR "general medicine"[tw] OR "Accountable Care Organizations"[Mesh] OR "ACO"[ti] OR "ACOs"[tw] OR "ACO’s"[tw] OR "care organization"[tw] OR "care organisation"[tw] OR "care organizations"[tw] OR "care organisations"[tw] OR "Ambulatory Care Facilities"[Mesh:NoExp] OR "Community Health Centers"[Mesh:NoExp] OR "health center"[tw] OR "health centers"[tw] OR "health centre"[tw] OR "health centres"[tw] OR "Health Maintenance Organizations"[Mesh] OR "maintenance organization"[tw] OR "maintenance organisation"[tw] OR "maintenance organizations"[tw] OR "maintenance organisations"[tw] OR "HMO"[ti] OR "HMOs"[ti] OR "HMO’s"[ti] OR "MCO"[ti] OR "MCOs"[ti] OR "MCO’s"[ti] OR "managed care"[tw] OR "integrated care"[tw]*)

Filters: Date 2007-2019, Language English
