# Supplementary material for: A systematic review of risk stratification tools internationally used in primary care settings
Source: Health Sci Rep. 2021 Jul 23;4(3):e329. doi: 10.1002/hsr2.329 (PMC8299990; doi:10.1002/hsr2.329)
Supplement: Supplementary file 2 — Appendix S2. Supporting Information. [file HSR2-4-e329-s001.docx]

Supporting Information file 2: Overview of included articles. Study-population, Outcome measure, Risk stratification model, Study type, Country and Journal of publication are shown.

| **Reference** | **Population** | **Outcome** | **Model** | **Study type** | **Country** | **Journal** |
| --- | --- | --- | --- | --- | --- | --- |
| (Aguado et al., 2008) **^10^** | Patients from five primary care centres  n=65,630 | Explained variability in drug expenditures | Adjusted Clinical Groups | Validation study | Spain | BMC Health Services Research |
| (Akazawa, Imai, Igarashi, & Tsutani, 2010) **^35^** | Adults aged 65 years old or older  n=6,628 | Incidence, healthcare utilization and costs associated with Potentially Inappropriate Medication Use | Elixhauser Classification System, Modified Beers criteria | Application study | Japan | The American Journal of Geriatric Pharmacotherapy |
| (Beauchet et al., 2019) **^36^** | Community dwelling adults aged 80 years or older, who visited a participating general practice within the study period  n = 668 | Hospital admissions (6 month follow-up) | 6-item Brief Geriatric Assessment | Validation study | France | Maturitas |
| (Brilleman & Salisbury, 2013) **^15^** | Primary care population of general practice  n=95,372 | Three year mortality and consultation rate | Charlson Comorbidity Index, Adjusted Clinical Groups, Quality and Outcomes Framework | Validation and comparison study | UK | Oxford University Press |
| (Brilleman et al., 2014) **^14^** | Primary care population of general practice  n=86,100 | Primary health costs | Charlson Comorbidity Index, Adjusted Clinical Groups, Quality and Outcomes Framework | Validation and comparison study | UK | Journal of Health Economics |
| (Burton et al., 2009) **^33^** | Non-institutionalized Peoples Health (Managed Care Organization) managed care beneficiaries  n=20,612 | Morbidity level | Adjusted Clinical Groups | Application study | USA | The American Journal of Managed Care |
| (Chang et al., 2017) **^37^** | Primary care population of the Health Partners Network  n=43,097 | Effect of prescription fill rates on risk stratification model performance | Adjusted Clinical Groups | Comparison study | USA | Medical Care |
| (Charlson et al., 2008) **^18^** | Primary care population of a general practice in an academic hospital  n=5,861 | Predict costs of disease in primary care patients | Adapted Charlson Comorbidity Index | Validation study | USA | Journal of Clinical Epidemiology |
| (Charlson et al., 2014) **^19^** | Medicaid managed care beneficiaries, adults and children, who received primary care in a specific medical center, not including Medicare/Medicaid patients (In this review presented results concern adult population)  n = 4,614 (2,218 adults and 2,396  children) | Identification of potential high cost beneficiaries | Charlson Comorbidity Index | Application study | USA | BMC Health Services Research |
| (Chung, Romanelli, Stults, & Luft, 2018) **^38^** | Medicare beneficiaries, aged 65 to 85 years, who were primary care patients in a large, mixed payer outpatient healthcare organization  n=108,734 | Preventive primary care visits | Own model including Charlson Comorbidity Index as comorbidity measure, age category, visit frequency and insurance | Application study | USA | Preventive Medicine |
| (Crane et al., 2010) **^39^** | Older community dwelling adults of a general practice  n=12,650 | High-Risk Hospitalization or ED admission | The Elders Risk Assessment | Validation study | USA | BMC Health Services Research |
| (Davis et al., 2018) **^40^** | Patients from an integrated healthcare delivery system and health plan, with continuous coverage; n = 2,118,343 | Health care costs | Hierarchical Condition Categories | Application study | USA | Journal of General Internal Medicine |
| (Dennis et al., 2019) ^41^ | Patients with type 2 diabetes who had been treated at a general practice  n = 161,575 | Hospital admission | Own prediction model | Validation study | Australia | BMC Health Services Research |
| (Duenas-Espin et al., 2016) **^42^** | Primary care populations  n=2,100,000 (B)  n=7,500,000 (C)  n=100,000) (L)  n=3,400,000 (Sc) | Health risk assessment | Adjusted Clinical Groups, Adjusted Morbidity Groups known as GMA, Clinical Risk Group | Application and comparison study | Spain, Italy, Scotland | BMJ Open |
| (Freund, Kunz, Ose, Szecsenyi, & Peters-Klimm, 2012) **^43^** | Primary care population  n=6,026 | Prediction risk future hospitalization | Hierarchical Condition Categories | Application study | Germany | Population Health Management |
| (Glazier, Agha, Moineddin, & Sibley, 2009) **^44^** | Primary care population  n=25,558 | Diagnosed health status | Adjusted Clinical Groups | Application study | Canada | Annals of Family Medicine |
| (Haas et al., 2013) **^4^** | Adult patients empaneled in 2009 and 2010 in a primary care practice  n=83,187 | Hospitalization, emergency department visits, 30-day readmission, high expenditures | Adjusted Clinical Group, Hierarchical Condition Categories, Elder Risk Assessment, Chronic Comorbidity Count, Charlson Comorbidity Index, Minnesota Tiering | Comparison study | USA | The American Journal of Managed Care |
| (Hamano, Oishi, & Kizawa, 2019) **^45^** | Primary care patients aged 65 years or older, who visited a participating general practice within the study period; n = 382 | Detoriation and dead | Supportive and Palliative Care Indicators Tool | Validation study | Japan | Journal of Pain and Symptom Management |
| (Hewner, Seo, Gothard, & Johnson, 2014) **^46^** | Primary care population of Medicare, Medicaid and privately insured  n=477,407 | Risk-stratified cohorts based on chronic disease and complexity | COMPLEXedex clinical algorithm | Application study | USA | Nursing Outlook |
| (Hong et al., 2015) **^47^** | Primary care adult patients in a practice-based research network  n=143,372 | Prediction of complexity | Outpatient Charlson Score & Commercial Risk Prediction | Validation and comparison study | USA | Journal of General Internal Medicine |
| (Hu et al., 2017) **^48^** | Primary care population  n=265 | Predictors of frequent visits to family physicians | Charlson Comorbidity Index, Beers Criteria | Application study | Canada | Canadian Familiy Physician |
| (Huntley, Johnson, Purdy, Valderas, & Salisbury, 2012) **^30^** | Primary care population | Care utilization, costs, mortality, quality of life | Adjusted Clinical Groups, Charlson Comorbidity Index, Chronic Disease Score, Cumulative Illness Rating Scale, Duke Severity Index | Review | - | Annals of Family Medicine |
| (Hutchings et al., 2013) **^49^** | Primary care population  n=2,400 | Estimate effects on the delivery of care, patient satisfaction, quality of life and resources used. | An emergency admission risk prediction tool called PRISM | Validation study | Wales | Trial Journal |
| (Inouye et al., 2008) **^20^** | Community dwelling elderly aged 70 years and older in primary care clinics of an academic medical center  n=3,919 | Unplanned hospitalization in one year | Deyo-Charlson, | Validation study | USA | Medical Care |
| (Khan et al., 2010) **^24^** | Primary care cancer patients and healthy controls  n=146,441 | Mortality | Adapted Charlson Score for use with Read/OXMIS instead of ICD10 diagnosis codes | Validation study | UK | BMC Family Practice |
| (Khanna et al., 2019) **^50^** | Patients who attended their primary care clinics at least once  n = 393,229 | 1 year hospitalization, ED visit | Own model | Validation study | Australia | Scientific Reports |
| (Kristensen et al., 2013) **^32^** | Primary care patients with type 2 diabetes;  n = 6,706 | Fee for service costs | Adjusted Clinical Groups | Application study | Denmark | Health Policy |
| **(Lemke et al., 2012) ^12^** | Primary care population  n=4,700,000 | Predicting hospitalization | Adjusted Clinical Groups, Charlson Comorbidity Index | Validation study | USA | Medical Care |
| (Maltenfort et al., 2019) **^11^** | Children seen in a large primary and specialty care outpatient network  n= 920,051 (70% for training and 30% for testing the model) | Unplanned 30-day hospitalization | Own model with predictors derived from the Adjusted Clinical Groups | Validation study | USA | PLoS One |
| (Martin et al., 2017) **^51^** | Patients from three primary care clinics participating in the Integrated Care Coordination Information System study data set, who had at least one of the selected conditions and were seen from 2008 to 2012  n = 750 | ED visits, hospitalization and heathcare costs | A modified Charlson Comorbidity Index, Hierarchical Condition Categories, count of chronic conditions defined by Affordable Care Act | Application study | USA | Applied Clinical Informatics |
| (Martin Lesende et al., 2018) **^52^** | Patients aged 65 years and older  n = 241 | Top 5% of the ‘Kaiser Permanente pyramid' | Adjusted Clinical Groups | Application study | Spain | BMJ Open |
| (Metcalfe et al., 2019) **^53^** | Patients from primary care practices: age- and sex-matched controls (n=26,860) for hip fractured patients (n = 13,974) | Mortality: 30-day and 1-year | Charlson Comorbidity Index, Elixhauser method | Validation study | UK | BMC Medical Research Methodology |
| (Milla-Perseguer, Guadalajara Olmeda, Vivas-Consuelo, & Uso-Talamantes, 2019) **^54^** | All citizens registered in a specific health district  n = 32,667 | Morbidity measure | Clinical Risk Groups | Application study | Spain | Health and Quality of Life Outcomes |
| (Moran et al., 2017) **^55^** | Primary care population (Medical University)  n=10,408 | Clustering patients with stratification on risk for hospital and ED utilization | Own model | Validation study | USA | Journal of Evaluation in Clinical Practice |
| **(Mosley et al., 2009) ^25^** | Primary care population, Medicare beneficiaries; n = 4,506 | Hospitalization | Hierarchical Condition Categories | Application study | USA | Journal of the American Geriatrics Society |
| (Muratov et al., 2019) **^56^** | Adults aged 66 years old or more  High cost users (n=175,847) and age and sex matched non-high cost users (n=527,541) | Hospital admission | Adjusted Clinical Groups | Validation study | Canada | CMAJ Open |
| (Noyes, Liu, & Temkin Greener, 2008) **^57^** | Community dwelling (not institutionalized for 90 days at a time) Medicare beneficiaries with continuous part A and B enrollment for at least two calender years  n = 46,790 | Healthcare Costs | Hierarchical Condition Categories | Validation study | USA | American Journal of Managed Care |
| (Ou et al., 2011) **^21^** | Medicaid enrolees with type 2 diabetes  n=9,832 | Health care behaviors (physician’s diabetes adherence standard adherence, patient’s medication adherence), health care utilization and expenditures | A modified version of Romano-adapted Charlson index, Elixhauser index, Chronic Disease Score, Health-related Quality of Life Comorbidity Index | Validation and Comparison study | USA | Health Outcomes Research in Medicine |
| (Ou et al., 2012) **^23^** | Medicaid enrolees with type 2 diabetes  n=9,832 | Health care utilization and expenditures | A modified version of Romano-adapted Charlson index, Elixhauser index, Chronic Disease Score, Health-related Quality of Life Comorbidity Index | Validation and Comparison study | USA | Population Health Management |
| (Ranstad, Midlov, & Halling, 2018) **^58^** | Primary care listed patients  n = 151,731 | Hospitalization (binary) and number of days hospitalized | Own model with doctor-patient relationship, contribution of complex diagnosis patterns (psychiatric disorders) and morbidity burden (Adjusted Clinical Groups based) | Application study | Sweden | Scandinavian Journal of Primary Health Care |
| (Rohrer, Rasmussen, & Adamson, 2008) **^59^** | Primary care population  n=698 | High utilization, Illness severity | Charlson Comorbidity Index | Application study | USA | Journal of Evaluation in Clinical Practice |
| (Salisbury, Johnson, Purdy, Valderas, & Montgomery, 2011) **^60^** | Primary care population  n=99,997 | Multimorbidity | Adjusted Clinical Groups, Quality of Outcome Framework | Application study | UK | British Journal of General Practice |
| (Shadmi et al., 2011) **^16^** | Adult enrolees of Clalit Health Services, Israel’s largest health care organization  n = 279,241 | Numbers of (1) primary care encounters, (2) specialist visits, (3) diagnostic imaging tests and (4) hospitalizations | Adjusted Clinical Groups, Charlson Comorbidity Index | Application, validation and comparison study | Israel | BMC Public Health |
| (Sibley, Moineddin, Agha, & Glazier, 2010) **^61^** | Primary care patients  n=25,558 | Predicting physician utilization | Adjusted Clinical Groups | Application study | Canada | Medical Care |
| (Sibley & Glazier, 2012) **^62^** | Primary care data (family health networks) n=487,131 | Expected healthcare utilization | Adjusted Clinical Groups | Application study | Canada | Health Policy |
| (Sicras-Mainar et al., 2007) **^63^** | Patients attending five primary care teams  n = 81,335 | Referral Rate | Adjusted Clinical Groups | Application study | Spain | European Journal of Public Health |
| (Sicras-Mainar et al., 2012) **^64^** | Patients from 13 primary care teams (86,5% primary care, 13,5% peadiatrics)  n = 227,235 | Heatlh costs | Adjusted Clinical Groups | Application study | Spain | BMJ Open |
| (Sicras-Mainar et al., 2013) **^13^** | Patients from 13 primary care teams  n = 227,235 | Explaining variance of: Visits, number of diagnoses, total costs | Adjusted Clinical Groups | Validation Study | Spain | Journal of Evaluation in Clinical Practice |
| (Sino et al., 2013) **^65^** | Hospitalized (cases) and matched non-hospitalized patients from pharmacy registries  n = 2 x 8,681 (cases and controls) | Prediction of hospital admission | Combining Prescription Changes Frequency, Chronic Disease Score | Application study | the Netherlands | BMC Pharmacology and Toxicology |
| (Snooks et al., 2019) **^66^** | Patients from primary care practices  n = 230,099 | Unscheduled hospital admission | An emergency admission risk prediction tool called PRISM | Application study | UK | BMJ Quality & Safety |
| (Soto-Gordoa et al., 2019) **^34^** | Patients with multimorbidity (at least two out of three conditions: diabetes, heart failure and chronic obstructive pulmonary disease) aged 65 years or more  n = 4,225 | Hospital admission | Adjusted Clinical Groups | Application study | Spain | Health Services Research |
| (Sternberg et al., 2012) **^67^** | Community dwelling elderly  n=221 | Predict hospitalizations and emergency department visitation | Adjusted Clinical Groups, Vulnerable Elderly Survey | Application study | Israel | American Journal of Managed Care |
| (Takahashi et al., 2013) **^68^** | Primary care population ECH Mayo clinic biobank  n=22,916 | Predict hospitalizations and emergency department visitation | Minnesota Tiering | Application study | USA | Mayo Clinic Proceedings |
| (Vest et al., 2019) **^69^** | Patients from primary care clinics linked to a public hospital: intervention group using risk stratification modelling (n=62,254) versus control group  n = 175,833 | Risk scores for needing a referral to different wraparound services such as behavioural health services, dietitian councelling and social services. | A machine learning algorithm, Elixhauser score | Application study | USA | The American Journal of Preventive Medicine |
| (Violan et al., 2013)**^70^** | Primary care population of 13 Primary Healthcare Centers  n=196,593 | Efficiency and effectiveness indicators on resource consumption | Adjusted Clinical Groups | Application study | Spain | BMC Health Service Research |
| (Vivas-Consuelo et al., 2014) **^71^** | Primary care population of general practice  n=261,054 | Predict pharmaceutical spending | ATC model*, Clinical Risk Groups | Application study | Spain | Health Policy |
| **(Vuik, Mayer, & Darzi, 2016) ^72^** | Primary and Secondary care population; n=300,000 | Identification high risk patients | Create risk scores, no tools mentioned | Validation study | UK | BMJ Open |
| **(Wallace et al., 2016) ^22^** | Older community dwelling adults aged 70 years or older  n = 862 | Predicting emergency hospital admission | Total disease count, Selected conditions disease count, Charlson Comorbidity Index, number of dispensed medication classes, RxRisk-V | Comparison study | Ireland | BMJ Open |
| **(Wennberg et al., 2013) ^73^** | Medicare beneficiaries  n=5,153,877 | Predicted one-year mortality | Charlson Comorbidity Index, Lezzoni chronic condition count, Hierarchical Condition Categories | Application study | USA | BMJ |
| **(Xu, Williams-Livingston, Gaglioti, McAllister, & Rust, 2018) ^74^** | Patients aged 18 year and older, seen in an urban academic family medicine clinic over a two-year period; n=5,364 | Utilization (high costs) | Elixhauser | Application study | USA | Journal of Health Care for the Poor and Underserved |
| **(Zhou, Wong, & Li, 2014) ^75^** | Elderly aged 65 years and older  n = 91,189 | Mortality, hospitalization and costs of care | Senior Segmentation Algorithm | Validation study | USA | Permanente Journal |

**ATC model categorizes patients into nine categories based on Anatomic Therapeutical Chemical (ATC) codes for medications.*
